# Microbiological Assessment and Production of Ochratoxin A by Fungi Isolated from Brazilian Dry-Cured Loin (Socol)

**DOI:** 10.3390/foods15030433

**Published:** 2026-01-24

**Authors:** Felipe Coser Chow, Gustavo Lucas Costa Valente, Viviana Patrícia Fraga Santos, Mariana Rodrigues Wenzel, Kelly Moura Keller, Carla Ferreira Soares, Henrique César Pereira Figueiredo, Marcelo Resende Souza, Silvana de Vasconcelos Cançado, Tadeu Chaves Figueiredo

**Affiliations:** Escola de Veterinária, Universidade Federal de Minas Gerais (UFMG), Avenida Antônio Carlos 6627, Belo Horizonte 30123-970, Brazil; felipecchow@gmail.com (F.C.C.); gustlcv.vet@gmail.com (G.L.C.V.); vivianafraga@yahoo.com.br (V.P.F.S.); rodrigues.mpaiva@gmail.com (M.R.W.); kelly.medvet@gmail.com (K.M.K.); carla.soares.vet@gmail.com (C.F.S.); figueiredoh@yahoo.com (H.C.P.F.); marceloresende51@gmail.com (M.R.S.); silvanacancado@gmail.com (S.d.V.C.)

**Keywords:** MALDI-TOF/MS, mycotoxins, microbiological quality, public health

## Abstract

This study aimed to evaluate the microbiological quality of Brazilian dry-cured loin (Socol), as well as the presence of ochratoxin A (OTA) and its synthesis by the isolated *Aspergillus ochraceus* complex under different conditions (culture media, temperature, and time of incubation). Nine bacterial genera were isolated and identified by Matrix Assisted Laser Desorption Ionization—Time Of Flight/Mass Spectrometry (MALDI-TOF/MS), including *Serratia* spp. (32.4%), *Citrobacter* spp. (20.9%), *Enterobacter* spp. (13.6%), and *Staphylococcus* spp. (6.3%), among others. *Salmonella* spp. was not observed, and counts of thermotolerant coliforms and coagulase-positive *Staphylococcus* were below the confidence level. Fifteen fungal strains were isolated and identified as *Aspergillus* spp. (n = 5), *Cladosporium* sp. (n = 1), and *Penicillium* spp. (n = 9). OTA was quantified in Socol samples, and the enumeration of fungi showed a correlation (r = 0.77) with the mycotoxin detection. *A. ochraceus* complex produced OTA in Czapek Yeast Autolyzed (CYA) and Yeast Extract Sucrose (YES) agars at different times and temperatures. It was concluded that the microbiota of Socol is complex, encompassing spoilage bacteria. Undesirable fungi are also present, including those belonging to the *A. ochraceus* complex that produce OTA.

## 1. Introduction

In recent years, consumers have shown a growing interest in “traditional or artisanal” foods. In general, this trend is explained by the perception of the consumers of these products as being associated with better quality, appearance, nutritional value, specific taste, healthiness, and safety. The dry-fermented meat products comprise hundreds of traditional variants, whose artisanal production has been transferred between generations as a practice to preserve meat during the year for family sustenance, as happens today in rural areas across the world. Most recently, these products have been manufactured on an artisanal scale in small manufacturing facilities in their geographical areas of origin [1].

The Brazilian dry-cured loin (Socol) is an artisanal meat product fermented by microorganisms present in the raw material and production environments. It is generally cured for at least 45 days. Socol originates from the Veneto region in Italy, and its production was introduced by Italian immigrants at the end of the 19th century in Venda Nova do Imigrante county (Espírito Santo, Brazil). Socol production represents a cultural heritage, and in 2018, it received the protected designation of origin [2]. The product is elaborated using commercial salt (NaCl, minimum 2.5%), black pepper (*Piper nigrum*), and garlic (*Allium sativum*). Then, the loin is wrapped in porcine peritoneum or artificial collagen-based wraps. During the curing period, filamentous fungi grow on the surface and develop lipolytic and proteolytic activities, which are related to the unique Socol flavor [3].

The pork is obtained from commercial confined swine (Landrace × Large White), raised in the area of protected designation of Socol origin and slaughtered in inspected slaughterhouses. Socol production begins with the portioning of the loin into three to four pieces, with or without trimming to remove part of the subcutaneous fat. Subsequently, the pieces are dry-salted for 48 h using salt with variable granulation. Excess salt is then removed from the surface by washing the pieces under running water, followed by seasoning. The meat is then stuffed into casings, and a starter culture is optionally inoculated. The stuffed pieces are placed in elastic netting, and hung on racks, where Socol is exposed to ambient conditions during the ripening process. The ripening stage is assessed by manual compression of the pieces, and once ripening is completed, the products are washed under running water again and brushed to remove surface molds. Finally, the products are hung again for drying for 12 to 48 h and subsequently packaged.

Since most cured raw products are stored without the need for refrigeration and are not subjected to any thermal treatment to ensure stability, the reduction in surface moisture and the action of various substances, either added as ingredients (salt, nitrite and/or nitrate, organic acids, and spices) or produced by lactic acid bacteria and fungi naturally occurring in raw materials or deliberately inoculated, are the main factors responsible for their long shelf-life. These aspects are directly related to the microbiological safety of the products, in addition to the quality control of raw materials and the adoption of good manufacturing practices [4].

Fungi make up the microbiota of raw and cured meat products. They contribute to their identity and sensory characteristics such as color, flavor, and texture [5,6]. On the other hand, cured meat products, like salami and raw hams, can be contaminated with undesirable fungi during processing or storage. This contamination can be caused by hygiene failures or the spread of mold in the production environment, especially inside the maturation chamber. Some species of fungi can produce toxic metabolites, such as ochratoxin A (OTA). The *Aspergillus ochraceus* complex (*Aspergillus* section Circumdati) is the name given to a set of species that has similar morphologic characteristics such as *A. melleus*, *A. ochraceus*, *A. ostianus*, *A. pallidofulvus*, *A. sesamicola*, and *A. westerdijkiae*, which are known to produce OTA [7].

Considering that there are few information on the literature about Socol, we hypothesize that this meat product may present variation in microbiological and physical-chemical parameters of quality, a diverse microbiota and the fungi found in this artisanal food may elaborate toxins leading to public health concern. Therefore, the objectives of this study were to evaluate the microbiological quality of Socol, isolate fungi, and search for the presence of OTA in Socol, as well as to study the OTA-producing potential of the isolated fungi in association with food innocuity.

## 2. Materials and Methods

### 2.1. Sampling

A total of 13 artisanal Socol samples produced at 10 different facilities were acquired from the local market in Venda Nova do Imigrante county, Espírito Santo State, Brazil (latitude 20°20′6″ S and longitude 41°07′49″ W). The Association of Socol Producers (Assocol) is composed of 25 members, including 10 families and 6 agro-industries [8]. Among the 10 producers from whom samples were collected, 7 were under some type of official inspection system (6 under municipal inspection and 1 under state inspection), while 3 were not inspected. During the sampling period, the climatic conditions of the region were characterized by temperatures varying from 16 to 26 °C (mean = 17 °C) and relative humidity ranging from 79 to 85% (mean = 82%). They were aseptically collected and transported under refrigeration (<5 °C) to the laboratory.

### 2.2. Physical Chemical Analyses

The determination of the moisture, protein, and lipid content of the Socol samples was carried out in triplicate according to the AOAC methodology [9].

### 2.3. Microbiological Analyses 

#### Isolation and Enumeration

Initially, 25 g of Socol was diluted in 225 mL of peptone saline (Oxoid, Basingstoke, Hampshire, England) and homogenized in a stomacher for 2 min. Decimal serial dilutions of up to 10^−6^ were made, and the sample were submitted to the counts of *Staphylococcus* spp., *S.* coagulase-positive, and coliforms. The plates were incubated and aerobiosis at 37 °C for 48 h [10].

The presence of *Salmonella* spp. was also investigated according to ISO 6579-1 [11]. Aliquots of 25 g of Socol were transferred to 225 mL of 1% peptone saline solution and incubated at 36 ± 2 °C for 18 h. Subsequently, 1 mL was transferred to 10 mL of Muller-Kauffmann Tetrathionate (MKTT) broth (Oxoid, Basingstoke, Hampshire, England) and incubated at 36 ± 2 °C for 24 h. Additionally, 0.1 mL was tr ansferred to 10 mL of Rappaport-Vassiliadis broth with soy (RVS) (Hexis, Jundiaí, São Paulo, Brazil) and incubated at 41.5 ± 2 °C for 24 h. After incubation, aliquots from both MKTT and RVS broths were streaked onto xylose lysine deoxycholate (XLD) agar (Titan Biotech, Rajasthan, India) and Brilliant Green Phenol-Red Lactose Sucrose (BPLS) agar (Oxoid, Basingstoke, Hampshire, England). The plates were incubated at 36 ± 2 °C for 24 h.

Colonies with different morphotypes grown in *Salmonella* and coliforms analyses were identified by Matrix Assisted Laser Desorption Ionization—Time Of Flight/Mass Spectrometry (MALDI-TOF/MS), using a Bruker Microflex MALDI Biotyper 2.0 (Bruker Daltonics, MA, USA), as previously described by Assis et al. [12]. The criterion recommended by the manufacturer was the following: a score ≥ 2000 indicates identification at the species level, <2000 and ≥1700 indicates identification at the genus level, and a score < 1700 is not reliable [13].

Fungi were isolated and enumerated on Dichloran Rose Bengal Chloramphenicol agar (DRBC) (Oxoid, Basingstoke, Hampshire, England). Ten grams of the sample were diluted in 90 mL of buffered peptone water (Oxoid, Basingstoke, Hampshire, England) and were agitated for five minutes. Decimal serial dilutions of up to 10^−4^ were made, and 100 µL was spread on the surface of the agar. Petri dishes were incubated under aerobiosis for seven days at 25 °C, and the fungi were enumerated at log CFU g^−1^ [14].

The identification of the fungi genera was carried out according to their macro and microscopic characteristics [15]. For each genus, the appropriate taxonomic key was used: Klich [16] for the genus *Aspergillus* and Pitt [17] for *Penicillium*.

The classification of *Aspergillus* spp. was based on the standard plating using three agars: Czapek Yeast Extract (CYA, Kasvi, Pinhais, Brazil), Czapek Yeast Extract 20% Sucrose (CY20S, Kasvi, Pinhais, Brazil), and Malt Extract (MEA, Kasvi, Pinhais, Brazil). Suspensions of conidia were prepared from each isolated fungus in 0.5 mL of semi-solid agar (0.2% agar-agar and 0.05% Tween 80). They were distributed in microtubes, a needle-shaped platinum loop was introduced into the conidia suspension, and three equidistant inoculation points were made in each medium. CYA plates were incubated for seven days at 25 °C and 37 °C, while MEA and CY20S were incubated for seven days at 25 °C.

The proposed key for *Penicillium* spp. was based on plating on CYA; MEA and 25% Glycerol Nitrate (G25N) agars. The preparation of the inoculum and inoculation on the culture media were similar to those used for *Aspergillus* spp. CYA plates were incubated for seven days at 5 °C, 25 °C, and 37 °C, while MEA and G25N were incubated for seven days at 25 °C.

During the identification of filamentous fungi isolated from Socol, microorganisms belonging to the *Aspergillus ochraceus* complex were detected. Therefore, the presence of ochratoxin A (OTA) in the Socol product was investigated, along with an in vitro evaluation of the OTA production potential by fungi isolated from Socol, given its public health relevance.

### 2.4. Ochratoxin A (OTA) Quantification in Socol

Twenty grams of Socol samples were weighed, without removing the casing, and 100 mL of a methanol:water solution (70:30 *v*/*v*) was added. Then, the samples were stirred at 1 g at 25 °C in an orbital shaker for one hour. Soon after, the samples were filtered using Whatman #1 filter. The filtrate was used for the detection and quantification of OTA by the Enzyme-Linked Immunosorbent Assay (ELISA), AgraQuant^®^ Ochratoxin 2/40 kits (Romer Labs Inc., Getzerstorf, Austria). The analyses were performed according to Biscotto et al. [18]. The samples were mixed with a mycotoxin–enzyme conjugate. Then, the mixture was transferred to antibody-coated microwells. After ten minutes, the wells were washed and enzyme substrate was added. The development of the blue color was observed. The color intensity was inversely proportional to the concentration of mycotoxin in the sample. The reaction was blocked with a stop solution, which changes the color from blue to yellow. Finally, the color intensity of each well was optically measured using an ELISA reader (Stat Fax 303 Plus Microstrip Reader, Awareness Technologies, Westport, CT, USA) with a 450 nm absorbance filter and a 630 nm differential filter. The optical densities (OD) of the samples were compared to the OD of standards through a linear regression in order to determine the concentration of OTA in each sample. Only calibration curves with R^2^ greater than 0.99 were considered. Analyses were performed in duplicate for each sample. The limit of detection (LOD) of the technique was 1.9 µg kg^−1^, and the limit of quantification (LOQ) was 2.0 µg kg^−1^.

### 2.5. In Vitro Evaluation of the OTA Production Potential by Fungi Isolated from Socol

Filamentous fungi isolated from Socol were evaluated in relation to their capacity for in vitro OTA production according to the methodology described by Frisvad et al. [19]. An aliquot of 20 mL of conidial suspension inocula (0.4 to 5.0 × 10^6^ CFU mL^−1^) adjusted by spectrophotometry (530 nm with an optical density reading of 80 to 82% transmittance) was inoculated on plates containing the following agars: Malt Extract (MEA, Kasvi, Pinhais, Brazil), Yeast Extract Supplemented (YES, Kasvi, Pinhais, Brazil), Dicloran Glycerol (DG18, Kasvi, Pinhais, Brazil), Creatine Sucrose (CREA, Kasvi, Pinhais, Brazil), Oat (OA, Kasvi, Pinhais, Brazil), and Czapek Yeast Autolysate (CYA, Kasvi, Pinhais, Brazil). All plates were incubated at 15 and 25 °C, with the exception of the CYA and YES, which were also incubated at 30 and 33 °C. This was followed by extraction based on Geisen et al. [20] with adaptations, in which three equidistant plugs were removed from each plate at 7, 14, and 21 days and transferred to microtubes, and 500 mL of chloroform was added. The microtubes were then centrifuged at 4000× *g* for 10 min and, subsequently, at 14,000× *g* for 5 min. Then, the supernatants were transferred to microtubes that were left open until the liquid extract was completely dry. They were frozen until analysis by High Performance Liquid Chromatography (HPLC) with a UV-Vis detector (Jasco LC-2000Plus HPLC system, Jasco Inc., Easton, MD, USA). At the time of use, the extracts were dissolved in 200 μL of the mobile phase. Detection and quantification of OTA from each sample were performed according to Scudamore and MacDonald [21]. Chromatographic separations were performed in reverse phase using a C18 column (Supelcosil™ LC-ABZ, Supelco, 150 × 4.6 mm, 5 μm particle size, St. Louis, MO, USA) connected to a pre-column (Supelguard LC-ABZ, Supelco; 20 × 4.6 mm, 5 µm particle size, St.Louis, MO, USA). The mobile phase consisted of acetonitrile:water:acetic acid (57:41:2 *v*/*v*/*v*), the injected volume was 10 μL, and the flow was 1.0 mL per minute. OTA detection was performed using wavelengths of 330 nm excitation and 460 nm emission. Peak area values were plotted against concentration, and calibration curves for OTA were constructed using least squares method for linear regression. The correlation coefficient and curve determination coefficient were 0.984 and 0.968, respectively. The LOD of 1.0 µg kg^−1^ was determined using the signal-to-noise ratio of 3:1, and the LOQ of 2.0 µg kg^−1^ was calculated as the minimum concentration using the signal-to-noise ratio of 10:1.

### 2.6. Statistical Analysis

The experiment was planned as a simple-sample design. The means and standard deviations were calculated and presented as a descriptive study.

## 3. Results and Discussion

### 3.1. Physical Chemical and Microbiological Analyses

The mean moisture, protein, and lipid content of Socol samples were 42.63%, 44.52%, and 11.79% (Table 1). The physicochemical quality of this fermented meat product is correlated with microbiological indicators and hygienic-sanitary assessment. The decrease in moisture during the ripening may limit the viability of microorganisms present in Socol. Also, the peptides and free fatty acids released during the ripening impair some bacterial metabolism, leading to their inhibition. Jimémez et al. [22] reported moisture values ranging from 38.42 to 41.39% and lipid contend similar in Iberian dry-cured loin. Regarding the protein content, similar results were also obtained in the present work in comparison with Seo et al. [23]. The amplitude can be explained by the artisanal way of producing the food, associated with the empirical method of determining the ideal curing point (digital compression).

No presence of *Salmonella* spp. was detected. Values were below the confidence limit for thermotolerant coliforms (<1.5 × 10^2^ CFU g^−1^) and coagulase-positive *Staphylococcus* (<2.0 × 10^2^ CFU g^−1^). This can be explained by the higher salt content and low water activity observed in ripened products such as Socol, which reduce microbial counts and result in the absence of pathogenic bacteria, thereby ensuring a safe product for consumption.

*Staphylococcus* spp. counts ranged from 4.1 to 7.7 log CFU g^−1^ (Table 2), which can be attributed to the ability of this microorganism to survive in environments with intermediate moisture levels, as is the case for short-ripened meat products like Socol. Unlike long-ripened dry-cured products, Socol undergoes a relatively short-ripened period, which may result in limited reductions in water activity and pH, creating favorable conditions for the survival of halotolerant bacteria such as *Staphylococcus* spp. Some members of this genus can withstand water activity (Aw) values as low as 0.85, as observed for the pathogenic species *S. aureus* [24]. According to Biscola et al. [25], halotolerant *Staphylococcus* spp. may remain viable in salted meat products. The presence of coagulase-negative staphylococci (CNS) may be beneficial for the sensory characteristics of Socol, as these bacteria may possess enzymatic systems (nitrate reductase, catalase, proteases, and lipases) that play important roles in the development of desirable color, flavor, and aroma in ripened meat products [26].

Socol producers monitor the ripening process by manually compressing the products, which may lead to the transfer of microorganisms naturally present on the hands of the handlers to the product. This digital compression is typically performed at approximately 45 days of ripening and highlights the importance of adopting Good Manufacturing Practices (GMPs) at this stage, particularly proper hand hygiene, to minimize the risk of microbial contamination. It is also important that producers perform this evaluation starting with the longest-ripened products and finishing with the less-ripened ones to avoid cross-contamination, as undesirable microbiota generally tend to decrease with longer ripening times. Rebecchi et al. [27] identified *Staphylococcus* spp. among the main microorganisms present in natural pork casings preserved in salt, which may indicate another potential source of these microorganisms in Socol, since 11 out of 13 agro-industries reported using porcine peritoneum as raw material. A common practice in the meat industry that could be adopted by producers is the decontamination of casings by immersion in organic acid solutions, such as acetic or lactic acid.

*Staphylococcus* spp. counts may pose a risk to consumers, since some strains are capable of producing staphylococcal enterotoxins. Although no coagulase-positive staphylococci were detected, coagulase-negative cocci have been identified as potential toxin producers [28]. Staphylococcal enterotoxin production may occur at temperatures ranging from 10 to 48 °C, pH from 4 to 9.6, and NaCl concentrations from 0 to 10% [29], conditions that can be found in Socol.

Only three Socol samples showed total coliform counts, two of which originated from the same inspected establishment and one from a non-inspected facility. These samples also exhibited elevated *Staphylococcus* spp. counts, which may suggest deficiencies in the implementation of GMP by both agro-industries. The low frequency and counts of coliforms were expected, as these microorganisms typically show low viability in low-water-activity environments such as that of Socol. Generally, total coliform and Enterobacteriaceae counts were lower in longer-ripened products [30]. To ensure the quality of Socol, these facilities should guarantee the hygienic handling of raw materials, prevent cross-contamination throughout the ripening process, maintain well-trained personnel, and improve the hygiene of the environment, equipment, and utensils.

The natural variability observed in microbiological and physicochemical analyses of artisanal meat products is inherent to the characteristics of this type of processing. Unlike industrial production, which operates under highly standardized and controlled conditions, artisanal systems typically rely on raw materials from small-scale producers, manual methods, and environmental conditions that are subject to oscillation in temperature, humidity, ventilation, and handling. These factors directly influence the chemical composition, microbiological behavior, and ripening dynamics of the product. Additionally, differences between batches, the equipment used, hygiene practices, processing time, and variations in the natural microbiota contribute to variabilities in analytical results. Therefore, a certain degree of variation is expected and considered normal within the context of artisanal products, reflecting the diversity of the production process and the inherent nature of these foods.

### 3.2. Proteomic Identification of Microorganisms Isolated from Socol

**Figure 1:**
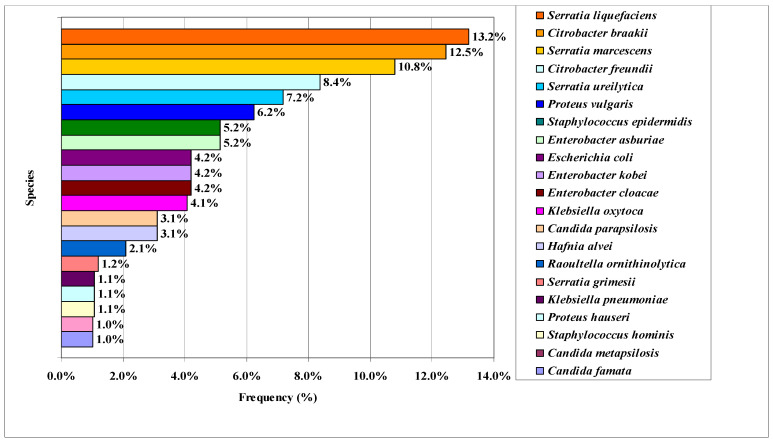
Frequencies of microorganisms isolated in Socol samples and identified by MALDI-TOF. (isolated from Baird Parker, XLD, or BPLS agars).

A total of 144 different morphotypes of microorganisms isolated from agar plates used for coliform and *Salmonella* sp. detection were subjected to MALDI-TOF MS analysis. Ninety-six isolates were identified, comprising species belonging to nine bacterial genera and one yeast genus (Figure 1). Bacteria belonging to the genera *Weissella* spp., *Pantoea* spp., and *Yarrowia* spp. were also detected. The most frequently identified genera included *Serratia* spp. (32.4%), *Citrobacter* spp. (20.9%), *Enterobacter* spp. (13.6%), and *Staphylococcus* spp. (6.3%), among others.

Most of the microorganisms identified belong to the family Enterobacteriaceae. These microorganisms act as spoilage agents and serve as indicators of hygienic conditions during production; however, a reduction in their counts is expected throughout the ripening of meat products. *Serratia liquefaciens*, *Hafnia alvei*, *Enterobacter cloacae*, and *Proteus vulgaris* are associated with the development of off-odors in dry-cured ham [31]. Spoiled bacteria like *S. liquefaciens* and *P. vulgaris* were reported in Serrano and Iberian hams, respectively, by Losantos et al. [32].

Similarly to the present study (Figure 1), Pasquali et al. [33] reported the presence of *Enterobacter cloacae*, *Escherichia coli*, *Citrobacter freundii*, and *Klebsiella pneumoniae* in artisanal Italian salami at 18 weeks and suggested that the ripening process was effective in reducing the risk associated with bacterial pathogens. *Citrobacter freundii*, *Citrobacter braakii*, *Staphylococcus hominis*, *Staphylococcus epidermidis*, *Proteus vulgaris*, *Klebsiella oxytoca*, *Klebsiella pneumoniae*, *Serratia marcescens*, *Raoultella ornithinolytica*, *Enterobacter cloacae*, *Enterobacter kobei*, *Enterobacter asburiae*, *Escherichia coli*, and *Candida metapsilosis* have also been reported in Brazilian artisanal salami [34].

Three *Candida parapsilosis*, one *Candida metapsilosis*, and one *Candida famata* (teleomorph *Debaryomyces hansenii*) were identified, in addition to six other isolates classified as *Candida* sp. *Candida* spp. are yeasts commonly found in fermented dry-cured products [35]. Mendoza et al. [36] reported *C. parapsilosis* in llama meat sausages from Argentina, and *C. famata* was isolated from dry-cured Parma ham [37]. *C. metapsilosis* and *C. parapsilosis* have been reported in Weining ham from China [38], and *C. metapsilosis* was isolated from pork sausages in Spain [39]. *Candida parapsilosis* is a common human commensal and one of the most frequently isolated fungi from human hands [40]. Yeasts exert important beneficial effects during ripening, as they contribute to the development of specific aroma and flavor through their proteolytic and lipolytic activities [41].

### 3.3. Enumeration and Identification of Fungi from Socol

The mean fungi counts in the Socol varied from 2.2 to 7.5 log CFU g^−1^ (Table 2). The presence of those microorganisms was not detected in four samples. Multz et al. [42] reported mean values of 3.6 log CFU g^−1^ of fungi in Socol. Fontana et al. [43] found fungi counts ranging from 4 to 5 log CFU g^−1^ in artisanal Argentine sausages, produced in the Andes region, using llama meat.

In the present work, the following fungi were isolated from Socol: *Cladosporium* sp. SFC1FA, *Penicillium camemberti* SFC2FA, *Aspergillus ochraceus* complex SFC5FA and SFC6FA, *Aspergillus fumigatus* complex SFC6FB, *Penicillium griseofulvum* SFC6FC, *Penicillium citrinum* SFC6FD, *Penicillium corylophilum* SFC7FA, *Penicillium citrinum* SFC7FB, *Aspergillus ochraceus* complex SFC7FC, *Penicillium roqueforti* SFC8FA, *Penicillium camemberti* SFC9FA, *Penicillium commune* SFC11FA, *Penicillium decumbens* SFC12FA, and *Aspergillus ochraceus* complex SFC12FB.

Fungi, such as *P. camemberti* and *P. roqueforti*, isolated in the present study, can contribute to the flavor of fermented meat products resulting from proteolysis processes [5,6]. However, undesirable fungi belonging to the *A. ochraceus* complex that produce mycotoxins, such as OTA, can represent public health problems [44].

### 3.4. Ochratoxin A (OTA) in Socol

**Figure 2:**
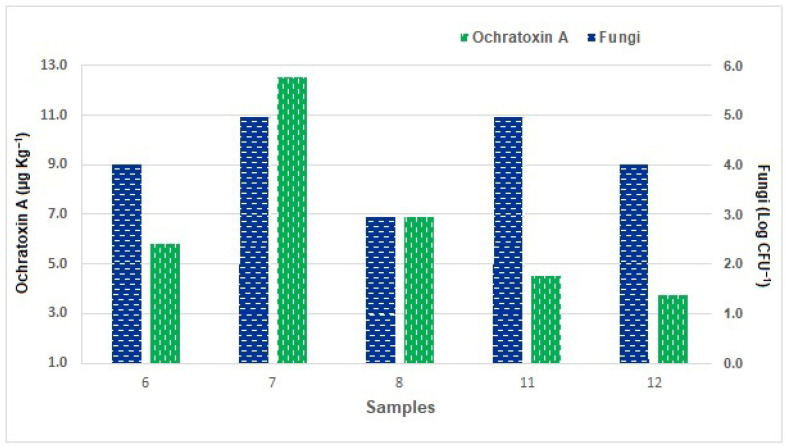
Fungi enumeration (Log CFU g^−1^) and ochratoxin A quantification (µg kg^−1^) in Socol samples collected at local market.

Of the 13 Socol samples analyzed, OTA was detected in 5 of them (6, 7, 8, 11, and 12) (Figure 2). Fungi counts ranged from 2 to 5 log CFU g^−1^ and the quantity of OTA from 3.72 to 12.53 µg kg^−1^. Fungi from the *A. ochraceus* complex were not present in Socol samples that did not show OTA detection (1, 2, 3, 4, 5, 9, 10, and 13). Although samples 1, 2, and 9 had fungi counts higher than 3 log CFU g^−1^, the presence of OTA was not detected. In two samples that were not contaminated with *A. ochraceus* complex, OTA was detected and quantified. A positive correlation (R = 0.77) was verified between the enumeration of fungi and the quantification of OTA in Socol.

In some Socol samples that did not contain OTA, the favorable conditions for its production may not have occurred due to the influence of moisture and salt concentrations. In addition, lactic acid bacteria are generally found in these products [45]. These microorganisms act as bioprotective agents by altering fungal viability and exerting an inhibitory effect, modulating the expression of genes involved in mycotoxin synthesis [46].

OTA is 1 µg kg^−1^ according to the Italian legislation [47]. Brazil, like the European Union and other countries, does not currently establish maximum limits for OTA in meat products. Regardless of the specific OTA limits established for this product category, regulations define maximum levels for this mycotoxin in other foods. Considering this fact, the OTA levels detected in the positive Socol samples exceeded the maximum limit (3 µg kg^−1^) permitted by the EU for processed cereals [48].

Probably, the explanation for the OTA levels detected in the present study is due to the presence of the casing in the analyzed samples. According to Iacumin et al. [49], OTA was found only on the sausage casings and was not detected in the interior. Moreover, the OTA concentration was higher in the outer than the inner parts of dry-cured ham samples [50].

Vipotnik et al. [44] reported that the production of OTA by fungi in cured and dehydrated meat products depends on factors such as water activity (aw), temperature, and the composition of the medium. Considering the fact that different batches of Socol are cured in a common environment, without moisture and temperature control, this may contribute to the dispersion of OTA-producing fungi. Furthermore, although the brushing and washing steps remove filamentous fungi and partially decrease OTA present on the surface of fermented sausages [51], they increase the risk of spreading fungi spores in the environment and on the products. So, brushing, washing, and removing the casing before consuming may be important to reduce the concentration of OTA, decreasing the potential risk to public health.

OTA is the main representative of the group of ochratoxins due to its toxicity. It is synthetized by several species of *Aspergillus* and *Penicillium*, mainly *A. carbonarius*, *A. niger* var. *niger*, *A. ochraceus*, *A. steynii*, *A. westerdijkiae*, *P. nordicum*, and *P. verrucosum* [44,52]. In animals, that toxin has immunotoxic, genotoxic, hepatotoxic, nephrotoxic, and mutagenic effects; furthermore, it is possibly carcinogenic [53]. In humans, OTA has been reported to cause nephropathy endemic to the Balkan region, a tumor-associated kidney disease [54].

The risk of OTA ingestion due to the consumption of cured meat products demonstrates the need to adopt preventive measures such as the use of non-toxigenic protective starter culture (*Debaryomyces hansenii*) or antifungal agents, coupled with the control of environmental conditions [55].

### 3.5. Production of Ochratoxin A (OTA) by Filamentous Fungi Isolated from Socol

**Figure 3:**
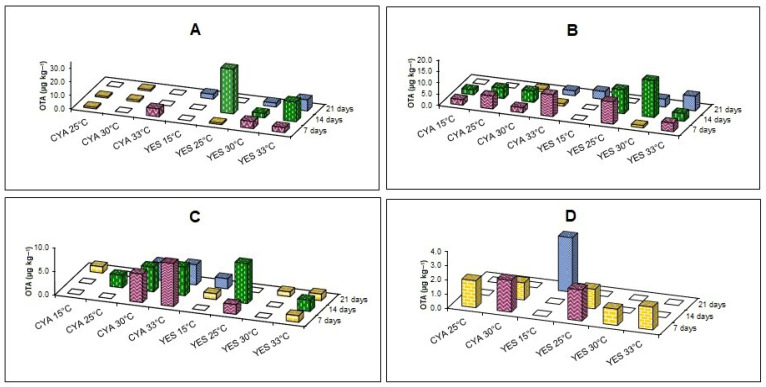
Effect of culture media (CYA and YES), time (7 days of incubation—red column, 14 days of incubation—green column, and 21 days of incubation—blue column), and temperature (15, 25, 30, and 33 °C) on ochratoxin A (OTA) production by *A. ochraceus* complex SFC5FA (**A**), SFC6FA (**B**), SFC7FC (**C**), and SFC12FB (**D**). LOQ < 1.9 µg kg^−1^ (yellow column) and LOD < 1.0 µg kg^−1^ (white column).

Four samples of fungi belonging to the *Aspergillus ochraceus* complex, isolated from Socol, were capable of producing OTA above the LOQ (2.0 µg kg^−1^) of the method on CYA and YES agars, when incubated at 15, 25, 30, and 33 °C for 7, 14, and 21 days (Figure 3).

The strains of *A. ochraceus* complex that produced the highest values of OTA were SFC5FA, incubated on YES at 25 °C for 14 days (32.83 µg kg^−1^) (Figure 3A); SFC6FA, on YES at 30 °C for 14 days (16.08 µg kg^−1^) (Figure 3B); SFC7FC, on CYA at 33 °C for 7 days (9.06 µg kg^−1^) (Figure 3C); and SFC12FB, on YES at 15 °C for 21 days (3.82 µg kg^−1^) (Figure 3D).

Therefore, fungi from the *A. ochraceus* complex present in the Socol microbiota have the potential to produce OTA. This fact confirms the risk to public health, as OTA was detected in Socol samples in quantities above the maximum limit permitted by the Italian legislation.

The results of OTA quantification in Socol, as well as in other foods, highlight the importance of controlling fungal growth and toxin production to ensure product safety and compliance with existing recommendations. In this context, the adoption of GMP and continuous training of food handlers are essential to ensure the safety and commercialization of these products.

## 4. Conclusions

Brazilian dry-cured loin (Socol) exhibits a complex and diverse microbiota, reflecting its artisanal nature, which likely contributed to the variability observed in the microbiological and physicochemical parameters. This wide range of results may also be explained by the limited sampling due to the small number of Socol-producing facilities. Among the bacterial species identified, spoilage microorganisms were detected, indicating the need for improvements in GMP. Fungi are also present in significant quantities in Socol, including desirable species contributing to flavor development and undesirable ones, such as members of the *Aspergillus ochraceus* complex. Since both OTA-producing fungi and the metabolite itself were detected, continuous monitoring of the Socol production process is required to prevent contamination by undesirable microorganisms and the formation of toxins that may pose risks to public health.

## Figures and Tables

**Table 1:** Means and standard deviations of physical chemical parameters in Socol samples collected at local market.

Analyses	Means	S.D.	Min	Max
Moisture	42.63	4.34	33.79	48.06
Lipid content	11.79	7.20	3.52	30.20
Protein	44.52	5.56	31.3	51.19

S.D.: standard deviations; Min: minimum; Max: maximum.

**Table 2:** Means and standard deviations of microbiological (log CFU g^−1^) parameters in Socol samples collected at local market.

Analyses	Means	S.D.	Min	Max
*Staphylococcus* spp.	6.7	1.1	4.1	7.7
Total coliforms	1.6	0.9	1.2	4.2
Yeast and Molds	4.0	1.7	2.2	7.5

S.D.: standard deviations; Min: minimum; Max: maximum.

## Data Availability

The original contributions presented in the study are included in the article, further inquiries can be directed to the corresponding author.

## References

[1] Rossi F., Tucci P., Del Matto I., Marino L., Amadoro C., Colavita G. (2023). Autochthonous Cultures to Improve Safety and Standardize Quality of Traditional Dry Fermented Meats. Microorganisms.

[2] INPI (Instituto Nacional da Propriedade Industrial) (2018). Revista da Propriedade Industrial.

[3] Lima T.T.M., Hosken B.O., Venturim B.C., Lopes I.L., Martin J.G.P. (2022). Traditional Brazilian fermented foods: Cultural and technological aspects. J. Ethn. Foods.

[4] Leroy F., Scholliers P., Amillien V. (2015). Elements of innovation and tradition in meat fermentation: Conflicts and synergies. Food. Sci. Tech..

[5] Copetti M.V. (2019). Yeasts and molds in fermented food production: An ancient bioprocess. Curr. Opin. Food Sci..

[6] Ashaolu T.J., Khalifa I., Mesak M.A., Lorenzo J.M., Farag M.A. (2021). A comprehensive review of the role of microorganisms on texture change, flavor and biogenic amines formation in fermented meat with their action mechanisms and safety. Crit. Rev. Food Sci. Nutr..

[7] Visagie C.M., Varga J., Houbraken J., Meijer M., Kocsubé S., Yilmaz N., Fotedar R., Seifert K.A., Frisvad J.C., Samson R.A. (2014). Ochratoxin production and taxonomy of the yellow aspergilli (*Aspergillus* section *Circumdati*). Stud. Mycol..

[8] Lopes M.P., Teixeira S.C., Vieira L.H.S., Pereira L.L. (2019). Caracterização da Associação de Produtores de Socol como Arranjo Produtivo Local: Uma contribuição para a valorização do agronegócio artesanal. Entrepreneurship.

[9] Association Of Official Analytical Chemists (2012). Official methods of analysis of AOAC International.

[10] (2001). Bacteriological Analytical Manual (BAM).

[11] (2017). Microbiology of the Food Chain—Horizontal Method for the Detection, Enumeration and Serotyping of Salmonella.

[12] Assis G.B.N., Pereira F.L., Zegarra A.U., Taveres G.C., Leal C.A., Figueiredo H.C.P. (2017). Use of MALDI-TOF Mass Spectrometry for the fast identification of Gram-Positive fish pathogens. Front. Microbiol..

[13] Singhal N., Kumar M., Kanaujia P.K., Virdi J.S. (2015). MALDI-TOF mass spectrometry: An emerging technology for microbial identification and diagnosis. Front. Microbiol..

[14] Pitt J.I., Hocking A.D. (2009). Fungi and Food Spoilage.

[15] Samson R.A., Hoekstra E.S., Frisvad J.C. (2004). Introduction to Food- and Airborne Fungi.

[16] Klich M.A. (2002). Identification of Common Aspergillus Species.

[17] Pitt J.I. (1988). A Laboratory Guide to Common Penicillium Species.

[18] Biscoto G.L., Salvato L.A., Alvarenga E.R., Dias R.R.S., Pinheiro G.R.G., Rodrigues M.P., Pinto P.N., Freitas R.P., Keller K.M. (2022). Mycotoxins in Cattle Feed and Feed Ingredients in Brazil: A Five-Year Survey. Toxins.

[19] Frisvad J.C., Frank J.M., Houbraken J. (2004). New ochratoxin producing species of *Aspergillus* section Circumdati. Stud. Mycol..

[20] Geisen R. (1996). Multiplex Polymerase Chain Reaction for the Detection of Potential Aflatoxin and Sterigmatocystin Producing Fungi. Syst. Appl. Microbiol..

[21] Scudamore K.A., MacDonald S.J. (1998). A collaborative study of an HPLC method for determination of ochratoxin A in wheat using immunoaflinity column clean-up. Food Addit. Contam..

[22] Jiménez A., González-Mohino A., Rufo M., Paniagua J.M., Antequera T., Perez-Palacios T. (2022). Dry-cured loin characterization by ultrasound physicochemical and sensory parameters. Eur. Food Res. Technol..

[23] Seo J.K., Lee Y.S., Eom J.U., Yang H.S. (2024). Comparing Physicochemical Properties, Fatty Acid Profiles, Amino Acid Composition, and Volatile Compounds in Dry-Cured Loin: The Impact of Different Levels of Proteolysis and Lipid Oxidation. Food Sci. Anim. Resour..

[24] Shekar A., Babu L., Ramlal S., Sripathy M.H., Batra H. (2017). Selective and concurrent detection of viable *Salmonella* spp., *E. coli*, *Staphylococcus aureus*, *E. coli* O157:H7, and *Shigella* spp., in low moisture food products by PMA-mPCR assay with internal amplification control. LWT.

[25] Biscola V., Todorov S.D., Capuano V.S.C., Abriouel H., Gálvez A., Franco B.D.G.M. (2013). Isolation and characterization of a nisin-like bacteriocin produced by a *Lactococcus lactis* strain isolated from charqui, a Brazilian fermented, salted and dried meat product. Meat Sci..

[26] Qin Y., Li W., Zhang W., Zhang B., Yao D., Zeng C., Cao J., Li L., Huang R. (2024). Characterization the microbial diversity and metabolites of four varieties of Dry-Cured ham in western Yunnan of China. Food Chem..

[27] Rebecchi A., Pisacane V., Miragoli F., Polka J., Falaskoni I., Morelli L., Puglisi E. (2015). High-throughput assessment of bacterial ecology in hog, cow and ovine casings used in sausages production. Int. J. Food Microbiol..

[28] Gundog D.A., Ozkaya Y., Gungor C. (2024). Pathogenic potential of meat-borne coagulase negative staphylococci strains from slaughterhouse to fork. Int. Microbiol..

[29] Savini F., Indio V., Giacometti F., Mekkonnen Y.T., De Cesare A., Prandini L., Marrone R., Seguino A., Di Paolo M., Vuoso V. (2024). Microbiological safety of dry-aged meat: A critical review of data gaps and research needs to define process hygiene and safety criteria. Ital. J. Food Saf..

[30] Bonilla-Luque O.M., Valero A., Tomasello F., Cabo M.L., Rodríguez-López P., Possas A. (2024). Exploring microbial diversity during the artisanal Salchichón production: Food safety in the consumer spotlight. LWT.

[31] Zhou C., Xia Q., Du L., He J., Sun Y., Dang Y., Geng F., Pan D., Cao J., Zhou G. (2022). Recent developments in off-odor formation mechanism and the potential regulation by starter cultures in dry-cured ham. Crit. Rev. Food Sci. Nutr..

[32] Losantos A., Sanabria C., Cornejo I., Carrascosa A.V. (2000). Characterization of Enterobacteriaceae strains isolated from spoiled dry-cured hams. Food Microbiol..

[33] Pasquali F., Valero A., Possas A., Lucchi A., Crippa C., Gambi L., Manfreda G., De Cesare A. (2023). Variability in Physicochemical Parameters and Its Impact on Microbiological Quality and Occurrence of Foodborne Pathogens in Artisanal Italian Organic Salami. Foods.

[34] Bastos M.M.F., Maran B.M., Maran E.M., Miotto-Lindner M., Verruck S. (2024). Assessment of microbial ecology in artisanal salami during maturation via metataxonomic analysis. Food Sci. Technol..

[35] Gong X., Mi R., Chen X., Zhu Q., Xiong S., Qi B., Wang S. (2023). Evaluation and selection of yeasts as potential aroma enhancers for the production of dry-cured ham. FSHW.

[36] Mendoza L.M., Padilla B., Carmela B.V., Vignolo G., De Las Rivas B. (2014). Diversity and enzymatic profile of yeasts isolated from traditional llama meat sausages from north-western Andean region of Argentina. Food Res. Int..

[37] Simoncini N., Rotelli D., Virgili R., Quintavalla S. (2007). Dynamics and characterization of yeasts during ripening of typical Italian dry-cured ham. Food Microbiol..

[38] Zhang S.Y., Tang N., Huang P., Zhou Y., Xu B.C., Li P.J. (2019). Isolation, identification and tolerance characteristics of microorganisms from Weining Ham. Meat Res..

[39] García-Béjar B., Árevalo-Villena M., Briones A. (2021). Characterization of yeast population from unstudied natural sources in La Mancha region. J. Appl. Microbiol..

[40] Kim J.H., Lee E.S., Kim B.M., Oh M.H. (2022). Potential Correlation between Microbial Diversity and Volatile Flavor Compounds in Different Types of Korean Dry-Fermented Sausages. Foods..

[41] Yang X., Xiao S., Wang J. (2024). *Debaryomyces hansenii* Strains from Traditional Chinese Dry-Cured Ham as Good Aroma Enhancers in Fermented Sausage. Fermentation.

[42] Mutz Y.S., Rosario D.K.A., Bernardo Y.A.A., Vieira C.P., Moreira R.V.P., Bernardes P.C., Conte-Junior C.A. (2022). Unravelling the relation between natural microbiota and biogenic amines in Brazilian dry-cured loin: A chemometric approach. Int. J. Food Sci. Technol..

[43] Fontana C., Bassi D., López C., Pisacane V., Otero M.C., Puglisi E., Rebecchi A., Cocconcelli P.S., Vignolo G. (2016). Microbial ecology involved in the ripening of naturally fermented llama meat sausages. A focus on lactobacilli diversity. Int. J. Food Microbiol..

[44] Vipotnik Z., Rodríguez A., Rodrigues P. (2017). *Aspergillus westerdijkiae* as a major ochratoxin A risk in dry-cured ham based-media. Int. J. Food Microbiol..

[45] Chow F.C., Valente G.L.C., Santos V.P.F., Soares C.F., Figueiredo H.C.P., Cançado S.d.V., Figueiredo T.C., Souza M.R. (2025). In Vitro Probiotic Potential of Lactic Acid Bacteria Isolated from Brazilian Dry-Cured Loin (Socol). Microorganisms..

[46] Li L., Yang B., Humza M., Geng H., Wang G., Zhang C., Gao S., Xing F., Liu Y. (2021). A novel strain *Lactobacillus brevis* 8-2B inhibiting *Aspergillus carbonarius* growth and ochratoxin a production. LWT.

[47] Sanità M.D., Italia Ministero della Sanità (1999). Circolare 09.06.1999. Gazzetta Ufficiale Repubblica Italiana.

[48] (2022). Commission Regulation (EU) 2022/1370 of 5 August 2022 amending Regulation (EC) No 1881/2006 as regards maximum levels of ochratoxin A in certain foodstuffs. Off. J. Eur. Union.

[49] Iacumim L., Chiesa L., Boscolo D., Manzano M., Cantoni C., Orlic S., Comi G. (2009). Moulds and ochratoxin A on surfaces of artisanal and industrial dry sausages. Food Microbiol..

[50] Bertuzzi T., Gualla A., Morlacchini M., Pietri A. (2013). Direct and indirect contamination with ochratoxin A of ripened pork products. Food Cont..

[51] Pleadin J., Staver M.M., Vahčić N., Kovačević D., Milone S.A., Saftić L., Scortichini G. (2015). Survey of aflatoxin B1 and ochratoxin A occurrence in traditional meat products coming from Croatian households and markets. Food Control..

[52] Gil-Serna P., García-Díaz M., González-Jaén M.T., Vázquez C., Patiño B. (2018). Description of an orthologous cluster of ochratoxin A biosynthetic genes in Aspergillus and Penicillium species. A comparative analysis. Int. J. Food Microbiol..

[53] EFSA CONTAM Panel (EFSA Panel on Contaminants in the Food Chain) (2020). Scientific opinion on the risk assessment of ochratoxin A in food. EFSA J..

[54] Stoev S.D. (2017). Balkan Endemic Nephropathy e Still continuing enigma, risk assessment and underestimated hazard of joint mycotoxin exposure of animals or humans. Chem-Biol. Interact..

[55] Roncero E., Andrade M.J., Álvarez M., Cebrián E., Rodríguez M. (2024). *Debaryomyces hansenii* reduces ochratoxin A production by *Penicillium nordicum* on dry-cured ham agar through volatile compounds. LWT.

